# Supplementary material for: Bird tolerance to humans in open tropical ecosystems
Source: Nat Commun. 2023 Apr 20;14:2146. doi: 10.1038/s41467-023-37936-5 (PMC10119130; doi:10.1038/s41467-023-37936-5)
Supplement: Supplementary file 1 — Supplementary information [file 41467_2023_37936_MOESM1_ESM.pdf]

Bird tolerance to humans in open tropical ecosystems

Supplementary Information

Peter Mikula*, Oldřich Tomášek, Dušan Romportl, Timothy K. Aikins, Jorge E. Avendaño, Bukola D. A. Braimoh-Azaki, Adams Chaskda, Will Cresswell, Susan J. Cunningham, Svein Dale, Gabriela R. Favoretto, Kelvin S. Floyd, Hayley Glover, Tomáš Grim, Dominic A. W. Henry, Tomas Holmern, Martin Hromada, Soladoye B. Iwajomo, Amanda Lilleyman, Flora J. Magige, Rowan O. Martin, Marina F. de A. Maximiano, Eric D. Nana, Emmanuel Ncube, Henry Ndaimani, Emma Nelson, Johann H. van Niekerk, Carina Pienaar, Augusto J. Piratelli, Penny Pistorius, Anna Radkovic, Chevonne Reynolds, Eivin Røskaft, Griffin K. Shanungu, Paulo R. Siqueira, Tawanda Tarakini, Nattaly Tejeiro-Mahecha, Michelle L. Thompson, Wanyoike Wamiti, Mark Wilson, Donovan R. C. Tye, Nicholas D. Tye, Aki Vehtari, Piotr Tryjanowski, Michael A. Weston, Daniel T. Blumstein & Tomáš Albrecht

This document contains Supplementary Methods, Figures, and Table.

*Correspondence to Peter Mikula (🖂 petomikula158@gmail.com)

Supplementary Methods

Predictor variables

Human disturbance

We used two indices of human disturbance at each site. First, each site was scored either as rural (0) or urban (1). Similar to the other studies on avian tolerance towards humans, we used a simple operational definition for urban sites as areas with continuous urban elements like multi-storey buildings, family houses or roads[1,2]. Rural sites were defined as areas with natural or agricultural landscapes with no or sparsely located houses. Human population density and the level of anthropogenic activity was found to be negatively correlated with avian flight initiation distance when there were harmless interactions with humans[1–3] and positively correlated when animals were harvested by humans[4–6]. For each study location, we obtained data on the global human footprint index (using a 2 km buffer radius) provided by SEDAC (available at https://sedac.ciesin.columbia.edu/data/set/wildareas-v3-2009-human-footprint)[7]. Human footprint index represents a cumulative index of human pressure estimated using eight variables including built-up environments, population density, electric power infrastructure, crop lands, pasture lands, roads, railways, and navigable waterways. We acknowledge that habitat type (rural or urban) is a more subjective proxy of human disturbance at sites than the human footprint index. However, habitat type is a long-established and widely-used proxy for the level of human disturbance in studies on escape behaviour of birds[1,8–10] and the two indexes may differ in some aspects of human disturbance they capture.

Starting distance

Starting distance is often positively correlated with flight initiation distance[5,11–13]; thus, the absence of knowledge on the starting distance may result in biased estimates of both the mean and variance in the flight initiation distance. We measured the starting distance (to the nearest metre) for each approached individual, defined as the distance between the initial position of human observer and the position of bird when first spotted and approached by an observer. The starting distance was estimated either by a number of ~1 m steps or using a rangefinder; all researchers using the "step method" were well-trained

before data collection to make their steps constantly ~1 m long or to be able to convert the distance measured by steps to metres, making these data directly comparable to data collected by rangefinders.

Body mass

Body size strongly predicts life-span and mortality of vertebrates[14,15]. Body size is one of the strongest life-history correlates of cross-species variation in the flight initiation distance; larger, longer-living birds escape typically earlier (i.e., have lower tolerance towards human approach) than do smaller species[1,16]. Body size was estimated as body mass (in grams); mean values of body mass were extracted from EltonTraits 1.0 database[17], largely using data from the compilation by ref.[18]. Body mass of *Sicalis columbiana* and *Paroaria nigrogenis* was collected from ref.[19] because of missing values in EltonTraits.

Clutch size

Clutch size is a life-history trait that reflects investment to current reproduction and may be associated with avian escape behaviour[11,20,21]. Adult birds are expected to tolerate greater risk (i.e. have lower tolerance towards humans) in species with more eggs in a single brood because the value of the current brood is high whereas adults of species with smaller clutches are expected to tolerate lower risk because the value of the current brood is lower[22]. Data on clutch size was assembled from ref.[19]; data for some species were obtained from alternative sources: *Chrysococcyx caprius* and *C. klaas* from ref.[23], *Crotophaga ani* from ref.[24], *Guira guira* from ref.[25], and *Tapera naevia* from ref.[26].

Migratory behaviour

Migratory birds were reported to have longer flight initiation distance than residents on wintering grounds[27,28]. Data on avian migratory behaviour were retrieved from BirdLife's digital database[29]. Migration was originally scored on a three-point scale: 1 = sedentary and nomadic (species moves in response to resources that are sporadic in time and distribution) species, 2 = altitudinal migrants (species makes regular / seasonal cyclical movements to higher / lower elevations with

predictable timing and destinations), and 3 = long-distance migrants (a substantial proportion of the global or regional species population makes regular / seasonal cyclical movements beyond the breeding range). However, because category "2" included only few species (N = 7), we rescored migration in following way: 0 = sedentary, nomadic species, and altitudinal migrant species, 1 = long-distance migrants.

Hand-wing index

Elongated wings are suitable for efficient long-distance flight and were found to be positively associated with avian flight initiation distance[30]. We collected data on the hand-wing index, a morphological metric used as a standard proxy for wing aspect ratio and enhanced avian flight performance[31–33], from ref.[34]. The hand-wing index was calculated as "100*D_K/L_w, where D_K is Kipp's distance (the distance between the tip of the first secondary feather and the tip of the longest primary feather) and L_w is wing length". Hence, resulting Kipp's distances were corrected for the wing length. High hand-wing index values indicate more elongated wings (high aspect ratio), whereas low hand-wing index values indicate less pointed/broader wings (low aspect ratio). The hand-wing index was found to increase with migration and increase at higher latitudes[34].

Ground foraging

Birds foraging on the ground have typically wide binocular vision which may enhance the detection and visual tracking of predators and facilitate food collection[35–37]. Ground foraging birds also spend significantly more time visually scanning their surroundings for predators than birds collecting food in vegetation and may engage in frequent head movements to monitor for potential risk[37]. Moreover, the risk of predation is highest near the ground[38,39] which may result in increased vigilance and earlier escape of ground foragers when compared with species foraging in other vegetation strata[40,41]. We thus calculated a ground foraging index which equals the proportion of foraging time spend on the ground or water when compared with the time spend elsewhere (understory, mid-story, canopy, and air) using data in EltonTraits 1.0 database[17].

Flock size

Flock size could influence risk perception in birds[42,43]. First, birds in larger flocks may escape earlier if prioritising reduction of risk and the decision of flocking animals to flee can be affected by the response of the most risk-sensitive individual in the flock[44–46]. Alternatively, birds may delay their escape with increasing flock size, suggesting that "dilution" or "many eyes" effects allow birds to maximize benefits of not escaping[12,21]. We estimated flock size as the number of all individuals feeding or roosting together in a group, visually separated from other birds. Flock size was estimated from the longer distance typically by using binoculars. Most of flock size estimates were taken from open habitats to minimize the effect of visibility. In most of case, we took data only for monospecific flocks; in a few instances and particularly in waterbirds, we collected data also for individuals that co-occurred in a flock with other species as these birds regularly formed mixed species flocks. However, different species permutations in mixed-species flocks resulted in the same escape distances in three out of five shorebird species[47].

Wet and dry season

Escape responses of birds may be season-dependant; for example, avian flight initiation distances were found to be longest during breeding season and declining toward non-breeding parts of annual life cycle in migratory birds breeding in Europe but wintering in tropical Africa[12]. However, breeding season for tropical birds is poorly known and, moreover, tropical species often exhibit temporal variation in the start and length of breeding season[48–51]. Nevertheless, we assigned each observation to the wet or dry season; for example, rainfall seasonality may cause temporal fluctuations in food resource abundance with effects on avian phenology (e.g., onset of breeding season)[52,53]. We examined average monthly rainfalls for each site and date on Climate Change Knowledge Portal (https://climateknowledgeportal.worldbank.org). We considered the wet season as months with the mean monthly average above the year-round mean, otherwise observations were assigned to the dry season.

Tree cover

Open tropical ecosystems differ significantly in contemporary tree cover[54,55]. Animals in less vegetated areas can detect predators at greater distances and, moreover, prey must move for longer distances to find a refugee in a protective cover which may result in longer flight initiation distances[56]. Alternatively, animals in less vegetated areas might delay their escape because they can monitor an approaching predator more easily than animals in dense habitats[21]. Because the local tree cover was usually not collected by us during the fieldwork, we estimated a tree cover within 2 km buffer radius around geographical position of each observation / site using data from Global Forest Watch (available at https://data.globalforestwatch.org/)[57]. This variable was used to control for large-scale variation in tree cover between sites rather than to estimate the effect of tree cover on FID in close proximity of approached individuals.

Continent, latitude, and altitude

Avian tolerance towards humans may differ between continents[58], and tend to show latitudinal variation, increasing from the Equator to the Poles[1,59] probably because of variation in predation risk and fecundity–survival trade-offs, and generally slower pace-of-life in tropical birds[22,60,61]. Avian tolerance in some bird species was also found to increase with altitude, similarly to latitudinal effects perhaps due to variation in adult or nest predation risk, but this relationship and its drivers are poorly understood[62,63]. Each observation was assigned to one of three continents – Africa, Australia, and South America, respectively. We converted the latitude coordinate of each site to its absolute value. Each site was also assigned to the altitude using data from United States Geological Survey (https://earthexplorer.usgs.gov/).

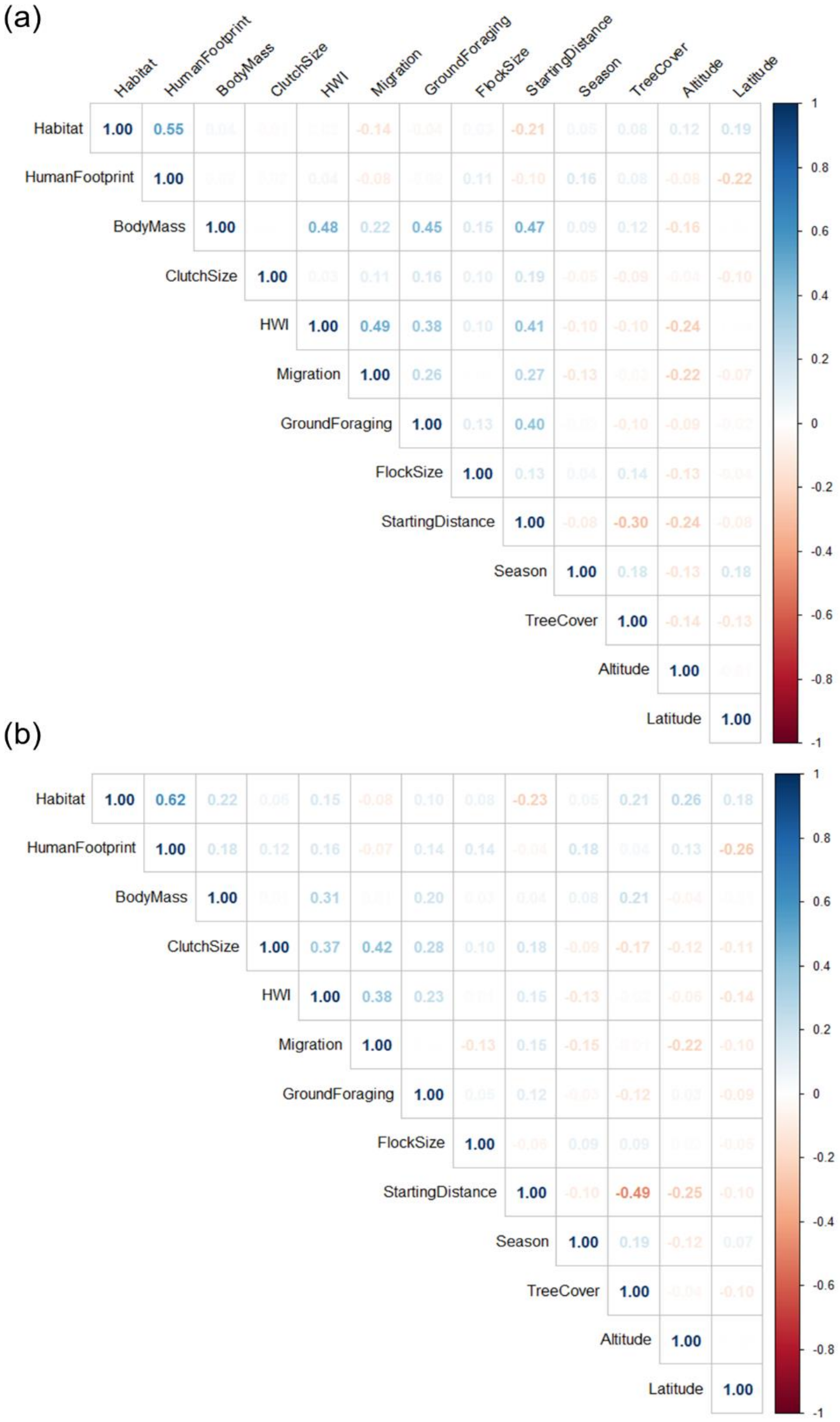

Supplementary Figure 1. Spearman's correlation coefficient matrix for continuous predictors used in the study. Correlations are provided for two datasets: (a) all species, and (b) passerine (Passeriformes) species only. Positive correlations are shaded by blue, negative correlations are shaded by red. The strength of the correlation is also indicated by colour saturation. Habitat – site-specific habitat type (urban/rural); HumanFootprint – site-specific human footprint; BodyMass – species-specific body mass; ClutchSize – species-specific clutch size; HWI – species-specific hand-wing index (wing shape); Migration – species-specific migratory status (sedentary, nomadic, altitudinal migrant species/long-distance migrants); GroundForaging – species-specific proportion of foraging time spend on the ground or water; FlockSize – individual-specific flock size; StartingDistance – individual-specific starting distance; Season – individual-specific season of data collection (wet/dry); TreeCover – site-specific percentage tree cover; Altitude – site-specific altitude; Latitude – site-specific absolute latitude. See method section and supplementary methods for further details on these variables.

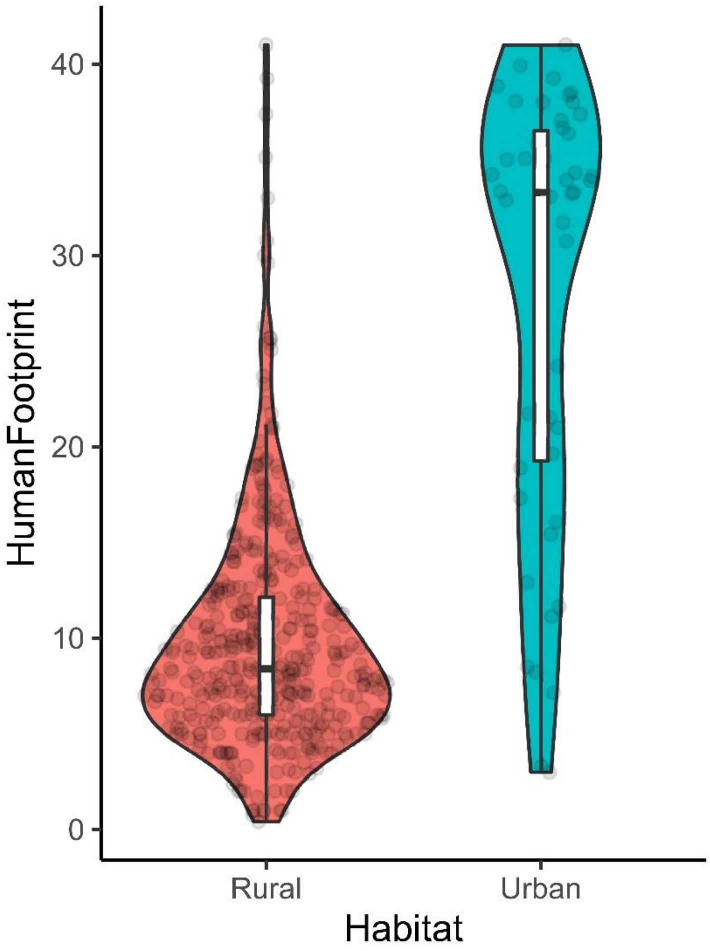

Supplementary Figure 2. The distribution of human footprint index for urban and rural sites. Boxplots are overlaid on a violin plots. Violin plots approximate the probability density of human footprint in each habitat type, while boxplots show the median (horizontal line in the middle of the boxes), upper (75%) and lower (25%) percentile (top and bottom of the box), and 1.5 times the inter-quartile range (whiskers). Displayed points represent human footprint values for each site.

Dataset	Habitat/footprint	Subset	R^2	Variable	Mean	Median	SD	MAD	2.5% CI	97.5% CI
All species	Both	No	0.1	Intercept	0.0094	0.0093	0.0475	0.046	-0.0838	0.1027
				**Habitat (urban)**	**-0.0674**	**-0.0678**	**0.0255**	**0.0253**	**-0.1178**	**-0.0184**
				**Human footprint**	**-0.055**	**-0.0553**	**0.0224**	**0.0227**	**-0.098**	**-0.0102**
				**Body mass**	**0.151**	**0.1511**	**0.0135**	**0.0135**	**0.1246**	**0.1771**
				**Clutch size**	**0.0198**	**0.0197**	**0.0094**	**0.0093**	**0.0016**	**0.0381**
				**Wing shape**	**0.0411**	**0.0411**	**0.0128**	**0.0128**	**0.0157**	**0.0662**
				Migration	0.0184	0.0183	0.0111	0.0113	-0.0034	0.0398
				Ground foraging	0.0077	0.0078	0.0103	0.0103	-0.0125	0.0281
				Flock size	-0.0022	-0.0022	0.0062	0.0063	-0.0142	0.01
				**Starting distance**	**0.6763**	**0.6764**	**0.0097**	**0.0098**	**0.6573**	**0.6954**
				**Season (wet)**	**0.0415**	**0.0415**	**0.0086**	**0.0085**	**0.0246**	**0.0586**
				Tree cover	-0.028	-0.0281	0.0264	0.0268	-0.0809	0.0218
				Continent (Australia)	-0.0489	-0.0495	0.0577	0.0576	-0.1618	0.066
				Continent (S America)	-0.0007	-0.0007	0.0568	0.057	-0.1117	0.1117
				Altitude	0.0163	0.0161	0.0217	0.022	-0.0264	0.059
				Latitude	-0.0268	-0.0273	0.0496	0.0497	-0.1223	0.0716
Passerines	Both	No	0.1	Intercept	-0.0182	-0.0186	0.0607	0.0588	-0.1383	0.1035
				**Habitat (urban)**	**-0.1128**	**-0.1118**	**0.0411**	**0.0416**	**-0.1954**	**-0.034**
				Human footprint	-0.0348	-0.0343	0.0357	0.0358	-0.1049	0.0362
				**Body mass**	**0.0681**	**0.0678**	**0.0192**	**0.0187**	**0.0312**	**0.1065**
				Clutch size	0.0235	0.0235	0.0178	0.0179	-0.0114	0.0582
				Wing shape	0.0274	0.0272	0.0187	0.0187	-0.0085	0.0644
				**Migration**	**0.0676**	**0.068**	**0.0231**	**0.0227**	**0.0224**	**0.113**
				Ground foraging	0.0084	0.0085	0.0168	0.0165	-0.0254	0.0413
				Flock size	0.0142	0.0142	0.0097	0.0097	-0.0049	0.0335
				**Starting distance**	**0.6829**	**0.6827**	**0.0138**	**0.0138**	**0.6564**	**0.7102**
				**Season (wet)**	**0.0394**	**0.0396**	**0.0145**	**0.0145**	**0.0112**	**0.0678**
				Tree cover	-0.0013	-0.0015	0.033	0.0328	-0.0676	0.0625
				Continent (Australia)	-0.0018	-0.0022	0.067	0.0675	-0.1321	0.131
				Continent (S America)	-0.0245	-0.0254	0.0634	0.0637	-0.1476	0.1014
				Altitude	0.0143	0.014	0.0297	0.0295	-0.044	0.0729
				Latitude	0.0001	-0.0003	0.0599	0.0589	-0.1167	0.1185
All species	Both	No	0.2	Intercept	0.0131	0.013	0.0479	0.0454	-0.0811	0.1094
				**Habitat (urban)**	**-0.0697**	**-0.0693**	**0.0259**	**0.0263**	**-0.1208**	**-0.0198**
				**Human footprint**	**-0.0559**	**-0.0562**	**0.0226**	**0.0222**	**-0.0995**	**-0.0108**
				**Body mass**	**0.1506**	**0.1507**	**0.0137**	**0.0135**	**0.1236**	**0.1779**
				**Clutch size**	**0.02**	**0.02**	**0.0093**	**0.0092**	**0.0016**	**0.0383**
				**Wing shape**	**0.0411**	**0.0411**	**0.0127**	**0.0128**	**0.0161**	**0.0657**
				Migration	0.0181	0.0181	0.0113	0.0115	-0.004	0.0401
				Ground foraging	0.0071	0.0072	0.0104	0.0102	-0.013	0.0274
				Flock size	-0.0025	-0.0025	0.0062	0.0062	-0.0149	0.0098
				**Starting distance**	**0.6807**	**0.6807**	**0.0097**	**0.0098**	**0.6615**	**0.6999**
				**Season (wet)**	**0.042**	**0.0419**	**0.0086**	**0.0087**	**0.0251**	**0.0586**
				Tree cover	-0.0298	-0.029	0.0275	0.0272	-0.0851	0.0241
				Continent (Australia)	-0.068	-0.0678	0.0696	0.0678	-0.2063	0.0675
				Continent (S America)	-0.0024	-0.0017	0.0657	0.065	-0.132	0.1307
				Altitude	0.0173	0.0172	0.0221	0.0222	-0.0255	0.0612
				Latitude	-0.0334	-0.0343	0.0548	0.0536	-0.1393	0.0775
Passerines	Both	No	0.2	Intercept	-0.0153	-0.0154	0.0653	0.0645	-0.1444	0.1129
				**Habitat (urban)**	**-0.1266**	**-0.1264**	**0.0447**	**0.0453**	**-0.2137**	**-0.0382**
				Human footprint	-0.0317	-0.0315	0.0385	0.0387	-0.1071	0.0427
				**Body mass**	**0.0683**	**0.0684**	**0.0194**	**0.0192**	**0.0298**	**0.1069**
				Clutch size	0.0231	0.0232	0.018	0.0181	-0.0121	0.0579
				Wing shape	0.0259	0.0256	0.0187	0.0188	-0.0112	0.0624
				**Migration**	**0.0706**	**0.0705**	**0.0236**	**0.0234**	**0.0246**	**0.117**
				Ground foraging	0.0083	0.0083	0.017	0.0173	-0.0245	0.0417
				Flock size	0.0138	0.0138	0.0098	0.0097	-0.0052	0.0333
				**Starting distance**	**0.6931**	**0.6931**	**0.0138**	**0.0139**	**0.6665**	**0.7202**
				**Season (wet)**	**0.0403**	**0.04**	**0.0145**	**0.0143**	**0.0121**	**0.0692**
				Tree cover	0	0.0002	0.035	0.0351	-0.0696	0.0685
				Continent (Australia)	-0.0034	-0.004	0.0825	0.0837	-0.1632	0.1582
				Continent (S America)	-0.0337	-0.0336	0.078	0.0765	-0.1861	0.119
				Altitude	0.0162	0.0165	0.0308	0.0305	-0.0427	0.0779
				Latitude	-0.002	-0.002	0.0717	0.0711	-0.1425	0.1422
All species	Both	No	0.3	Intercept	0.015	0.014	0.0495	0.0465	-0.0817	0.1162
				**Habitat (urban)**	**-0.0711**	**-0.0709**	**0.0268**	**0.0264**	**-0.1244**	**-0.018**
				**Human footprint**	**-0.0556**	**-0.0555**	**0.0233**	**0.0232**	**-0.101**	**-0.0097**
				**Body mass**	**0.1503**	**0.1503**	**0.0137**	**0.0137**	**0.1239**	**0.1771**
				**Clutch size**	**0.02**	**0.02**	**0.0093**	**0.0093**	**0.0019**	**0.0384**
				**Wing shape**	**0.041**	**0.0411**	**0.0128**	**0.0128**	**0.0157**	**0.0657**
				Migration	0.0179	0.0181	0.0112	0.0111	-0.0044	0.0394
				Ground foraging	0.0067	0.0067	0.0104	0.0102	-0.0135	0.0268

Table S1 continued

**Dataset**			**R^2**	**Variable**	**Mean**	**Median**	**SD**	**MAD**	**2.5% CI**	**97.5% CI**
				Flock size	-0.0026	-0.0025	0.0063	0.0062	-0.015	0.0096
				**Starting distance**	**0.6824**	**0.6825**	**0.0099**	**0.0098**	**0.6627**	**0.7022**
				**Season (wet)**	**0.042**	**0.0421**	**0.0085**	**0.0087**	**0.0256**	**0.0586**
				Tree cover	-0.0304	-0.0307	0.0284	0.0286	-0.0868	0.0245
				Continent (Australia)	-0.0773	-0.0762	0.0741	0.0741	-0.2268	0.0659
				Continent (S America)	-0.002	-0.0032	0.0716	0.0704	-0.141	0.138
				Altitude	0.0176	0.0175	0.0224	0.022	-0.0258	0.0624
				Latitude	-0.0378	-0.0382	0.0576	0.0567	-0.1509	0.0774
All species	Footprint	No	0.3	Intercept	0.0026	0.0024	0.0482	0.0464	-0.0925	0.0989
				**Human footprint**	**-0.0846**	**-0.0847**	**0.0209**	**0.0208**	**-0.125**	**-0.043**
				**Body mass**	**0.1497**	**0.1497**	**0.0136**	**0.0135**	**0.1227**	**0.1765**
				**Clutch size**	**0.02**	**0.0201**	**0.0094**	**0.0095**	**0.0018**	**0.0383**
				**Wing shape**	**0.0413**	**0.0413**	**0.0128**	**0.013**	**0.0162**	**0.0662**
				Migration	0.0175	0.0174	0.0112	0.0113	-0.0045	0.04
				Ground foraging	0.0065	0.0064	0.0103	0.01	-0.014	0.027
				Flock size	-0.003	-0.003	0.0062	0.0062	-0.015	0.0094
				**Starting distance**	**0.6829**	**0.6829**	**0.0096**	**0.0094**	**0.6638**	**0.7015**
				**Season (wet)**	**0.0418**	**0.042**	**0.0086**	**0.0086**	**0.025**	**0.059**
				Tree cover	-0.0317	-0.0318	0.028	0.0276	-0.0879	0.0231
				Continent (Australia)	-0.0787	-0.0791	0.0751	0.0744	-0.2241	0.0698
				Continent (S America)	-0.0007	-0.0012	0.0706	0.0703	-0.1377	0.1404
				Altitude	0.0191	0.0189	0.0226	0.0226	-0.0241	0.0633
				Latitude	-0.0358	-0.0361	0.0572	0.057	-0.147	0.0755
All species	Habitat	No	0.3	Intercept	0.0224	0.0218	0.0504	0.0491	-0.0743	0.121
				**Habitat (urban)**	**-0.0997**	**-0.0998**	**0.0239**	**0.0239**	**-0.1456**	**-0.0521**
				**Body mass**	**0.1502**	**0.1502**	**0.0136**	**0.0135**	**0.1238**	**0.177**
				**Clutch size**	**0.02**	**0.0199**	**0.0093**	**0.0095**	**0.0017**	**0.0382**
				**Wing shape**	**0.0402**	**0.0403**	**0.0127**	**0.013**	**0.0148**	**0.0646**
				Migration	0.0185	0.0185	0.0113	0.0114	-0.0033	0.0407
				Ground foraging	0.0073	0.0073	0.0104	0.0103	-0.0136	0.0272
				Flock size	-0.0025	-0.0024	0.0063	0.0064	-0.0149	0.0096
				**Starting distance**	**0.6826**	**0.6828**	**0.0098**	**0.0097**	**0.6631**	**0.7016**
				**Season (wet)**	**0.042**	**0.0419**	**0.0085**	**0.0087**	**0.0258**	**0.0586**
				Tree cover	-0.0258	-0.0255	0.0279	0.0278	-0.0798	0.0293
				Continent (Australia)	-0.0778	-0.0769	0.075	0.0738	-0.2253	0.0671
				Continent (S America)	-0.0053	-0.0066	0.0715	0.0703	-0.1435	0.138
				Altitude	0.012	0.0118	0.0221	0.0216	-0.0306	0.0566
				Latitude	-0.0302	-0.0302	0.0587	0.0583	-0.1442	0.0855
All species	Both	Yes	0.3	Intercept	0.0932	0.0934	0.0742	0.0681	-0.0537	0.2426
				**Habitat (urban)**	**-0.0996**	**-0.0996**	**0.0339**	**0.0339**	**-0.1656**	**-0.0342**
				Human footprint	-0.0471	-0.0473	0.0393	0.0394	-0.1228	0.0301
				**Body mass**	**0.1255**	**0.1253**	**0.0253**	**0.0255**	**0.0769**	**0.1753**
				**Clutch size**	**0.0456**	**0.0457**	**0.0185**	**0.0185**	**0.0093**	**0.0814**
				Wing shape	0.0279	0.0279	0.0246	0.0247	-0.0195	0.0758
				Migration	0.0371	0.037	0.0225	0.0229	-0.0065	0.0812
				**Ground foraging**	**-0.0473**	**-0.047**	**0.0212**	**0.0217**	**-0.0877**	**-0.0055**
				Flock size	0.0098	0.0097	0.0098	0.0098	-0.0093	0.0286
				**Starting distance**	**0.6501**	**0.6503**	**0.0156**	**0.0157**	**0.6192**	**0.6804**
				Season (wet)	0.0242	0.024	0.0141	0.0139	-0.0036	0.0525
				Tree cover	0.0081	0.0084	0.0386	0.0386	-0.0659	0.0844
				Continent (Australia)	-0.0079	-0.0099	0.0857	0.0835	-0.175	0.1675
				Continent (S America)	-0.0612	-0.0629	0.0846	0.0823	-0.2221	0.1162
				Altitude	0.0171	0.0167	0.0323	0.0321	-0.0459	0.0798
				Latitude	-0.0555	-0.0577	0.0662	0.0635	-0.1812	0.0821
				Intercept	0.0026	0.0024	0.0482	0.0464	-0.0925	0.0989
All species	Footprint	Yes	0.3	Intercept	0.0594	0.0611	0.0714	0.0666	-0.0851	0.1996
				**Human footprint**	**-0.0987**	**-0.0985**	**0.0357**	**0.0359**	**-0.1673**	**-0.0281**
				**Body mass**	**0.1247**	**0.1248**	**0.025**	**0.0248**	**0.0754**	**0.1744**
				**Clutch size**	**0.0452**	**0.0449**	**0.0184**	**0.0185**	**0.0087**	**0.0811**
				Wing shape	0.028	0.0277	0.0241	0.0239	-0.0183	0.0762
				Migration	0.0362	0.0362	0.0227	0.0222	-0.008	0.081
				**Ground foraging**	**-0.0486**	**-0.0484**	**0.0208**	**0.021**	**-0.0899**	**-0.0083**
				Flock size	0.0084	0.0085	0.0096	0.0094	-0.0106	0.0271
				**Starting distance**	**0.6516**	**0.6514**	**0.0158**	**0.0158**	**0.62**	**0.6829**
				Season (wet)	0.0242	0.0241	0.0141	0.014	-0.0032	0.0519
				Tree cover	0.0069	0.0071	0.0391	0.0388	-0.0708	0.085
				Continent (Australia)	-0.0045	-0.0057	0.0884	0.0861	-0.1771	0.1746
				Continent (S America)	-0.0542	-0.0574	0.0853	0.085	-0.2152	0.1195
				Altitude	0.0138	0.0139	0.0333	0.0334	-0.0502	0.0787
				Latitude	-0.0488	-0.0498	0.0675	0.0644	-0.1782	0.0917

Table S1 continued

**Dataset**			**R^2**	**Variable**	**Mean**	**Median**	**SD**	**MAD**	**2.5% CI**	**97.5% CI**
All species	Habitat	Yes	0.3	Intercept	0.1051	0.1053	0.0734	0.0682	-0.0475	0.2479
				**Habitat (urban)**	**-0.1177**	**-0.1177**	**0.0301**	**0.0299**	**-0.1773**	**-0.0574**
				**Body mass**	**0.1249**	**0.1249**	**0.0251**	**0.0245**	**0.0756**	**0.1745**
				**Clutch size**	**0.0456**	**0.0455**	**0.0187**	**0.0186**	**0.0092**	**0.0818**
				Wing shape	0.0281	0.0281	0.0242	0.0242	-0.0193	0.0761
				Migration	0.0376	0.0375	0.0224	0.0223	-0.0069	0.0816
				**Ground foraging**	**-0.0479**	**-0.0478**	**0.0206**	**0.0204**	**-0.0879**	**-0.0067**
				Flock size	0.0096	0.0096	0.0096	0.0097	-0.0089	0.0285
				**Starting distance**	**0.6505**	**0.6506**	**0.0158**	**0.0158**	**0.6197**	**0.681**
				Season (wet)	0.0234	0.0236	0.0138	0.0138	-0.0039	0.0504
				Tree cover	0.0105	0.0105	0.0386	0.0383	-0.0654	0.0859
				Continent (Australia)	-0.0054	-0.0065	0.0869	0.0834	-0.171	0.1719
				Continent (S America)	-0.0591	-0.0599	0.0843	0.0833	-0.2197	0.113
				Altitude	0.0167	0.0166	0.0328	0.0329	-0.0478	0.0813
				Latitude	-0.0542	-0.0559	0.0663	0.0641	-0.1812	0.0804
Passerines	Both	No	0.3	Intercept	-0.0125	-0.0127	0.0648	0.0621	-0.139	0.1165
				**Habitat (urban)**	**-0.1331**	**-0.1338**	**0.046**	**0.0459**	**-0.2238**	**-0.0431**
				Human footprint	-0.029	-0.0293	0.0393	0.0391	-0.1051	0.048
				**Body mass**	**0.0688**	**0.0686**	**0.0196**	**0.0195**	**0.0299**	**0.107**
				Clutch size	0.0234	0.0235	0.0178	0.0177	-0.0112	0.0584
				Wing shape	0.0248	0.0249	0.0191	0.0192	-0.0127	0.0619
				**Migration**	**0.0713**	**0.0711**	**0.0239**	**0.0242**	**0.0254**	**0.1174**
				Ground foraging	0.0079	0.0079	0.017	0.0168	-0.0254	0.0415
				Flock size	0.0139	0.014	0.0097	0.0097	-0.0057	0.0328
				**Starting distance**	**0.6963**	**0.6965**	**0.0141**	**0.0139**	**0.6688**	**0.7239**
				**Season (wet)**	**0.0405**	**0.0404**	**0.0146**	**0.0144**	**0.0119**	**0.0692**
				Tree cover	0.0018	0.002	0.0357	0.0363	-0.0679	0.0702
				Continent (Australia)	-0.0055	-0.0055	0.0914	0.0914	-0.1827	0.1725
				Continent (S America)	-0.0409	-0.0415	0.0863	0.086	-0.2091	0.1308
				Altitude	0.0171	0.0172	0.0311	0.0314	-0.0439	0.078
				Latitude	-0.0033	-0.0037	0.0779	0.077	-0.1567	0.1525
Passerines	Footprint	No	0.3	Intercept	-0.037	-0.038	0.0645	0.0636	-0.1632	0.0936
				**Human footprint**	**-0.0875**	**-0.0874**	**0.0344**	**0.0344**	**-0.1554**	**-0.0201**
				**Body mass**	**0.0673**	**0.0673**	**0.0197**	**0.0197**	**0.0286**	**0.1059**
				Clutch size	0.0231	0.0233	0.0179	0.0176	-0.0121	0.0586
				Wing shape	0.0267	0.0267	0.0188	0.019	-0.0097	0.0629
				**Migration**	**0.0712**	**0.0715**	**0.0236**	**0.023**	**0.0241**	**0.118**
				Ground foraging	0.0071	0.007	0.0169	0.0168	-0.0256	0.0398
				Flock size	0.0134	0.0133	0.0097	0.0098	-0.0051	0.0329
				**Starting distance**	**0.698**	**0.6981**	**0.014**	**0.014**	**0.6703**	**0.725**
				**Season (wet)**	**0.0408**	**0.0409**	**0.0145**	**0.0146**	**0.0128**	**0.0689**
				Tree cover	-0.0057	-0.005	0.0362	0.0364	-0.077	0.0641
				Continent (Australia)	-0.0017	-0.0024	0.0923	0.0904	-0.1814	0.181
				Continent (S America)	-0.0384	-0.0397	0.0863	0.0867	-0.2031	0.1294
				Altitude	0.0156	0.0152	0.0311	0.0308	-0.0454	0.0756
				Latitude	0.0036	0.0029	0.0773	0.076	-0.1481	0.1592
Passerines	Habitat	No	0.3	Intercept	-0.0087	-0.0092	0.0662	0.0652	-0.1385	0.1219
				**Habitat (urban)**	**-0.1511**	**-0.151**	**0.0395**	**0.0386**	**-0.2292**	**-0.074**
				**Body mass**	**0.0684**	**0.0686**	**0.0199**	**0.02**	**0.0296**	**0.1077**
				Clutch size	0.0236	0.0234	0.018	0.018	-0.0117	0.059
				Wing shape	0.0248	0.0246	0.0188	0.0187	-0.0113	0.0618
				**Migration**	**0.0713**	**0.0716**	**0.0236**	**0.0239**	**0.0245**	**0.1159**
				Ground foraging	0.0079	0.0076	0.0171	0.0172	-0.0252	0.0423
				Flock size	0.0138	0.0138	0.0097	0.0096	-0.005	0.033
				**Starting distance**	**0.6964**	**0.6966**	**0.0138**	**0.0137**	**0.6688**	**0.7235**
				**Season (wet)**	**0.0402**	**0.0401**	**0.0146**	**0.0144**	**0.0117**	**0.0689**
				Tree cover	0.0043	0.0046	0.0356	0.0356	-0.0636	0.0743
				Continent (Australia)	-0.0068	-0.0072	0.0916	0.0906	-0.1874	0.1753
				Continent (S America)	-0.0422	-0.0443	0.0863	0.0877	-0.2061	0.1294
				Altitude	0.0142	0.0143	0.0307	0.0301	-0.0454	0.0753
				Latitude	-0.0007	-0.0001	0.0771	0.0777	-0.1497	0.1487
All species	Both	No	0.5	Intercept	0.0165	0.016	0.0494	0.0475	-0.0812	0.115
				**Habitat (urban)**	**-0.0716**	**-0.0712**	**0.0269**	**0.027**	**-0.1251**	**-0.0202**
				**Human footprint**	**-0.056**	**-0.0557**	**0.0234**	**0.0235**	**-0.1016**	**-0.0116**
				**Body mass**	**0.1507**	**0.1506**	**0.0138**	**0.0139**	**0.1237**	**0.1776**
				**Clutch size**	**0.0201**	**0.0201**	**0.0092**	**0.0093**	**0.002**	**0.038**
				**Wing shape**	**0.0405**	**0.0405**	**0.0127**	**0.0129**	**0.0161**	**0.0651**
				Migration	0.0181	0.0181	0.0112	0.0111	-0.0032	0.0403
				Ground foraging	0.0067	0.0067	0.0104	0.0103	-0.0135	0.0275
				Flock size	-0.0027	-0.0027	0.0062	0.0063	-0.015	0.0094
				**Starting distance**	**0.6834**	**0.6834**	**0.0096**	**0.0097**	**0.6646**	**0.7022**
				**Season (wet)**	**0.0422**	**0.0424**	**0.0087**	**0.0087**	**0.0249**	**0.0589**

Table S1 continued

Dataset			R^2	Variable	Mean	Median	SD	MAD	2.5% CI	97.5% CI
				Tree cover	-0.0313	-0.0315	0.0279	0.0276	-0.0855	0.0241
				Continent (Australia)	-0.0878	-0.086	0.0788	0.0773	-0.2465	0.0649
				Continent (S America)	-0.0049	-0.0059	0.0747	0.074	-0.1541	0.1429
				Altitude	0.0174	0.0173	0.0226	0.0227	-0.0264	0.061
				Latitude	-0.0417	-0.0416	0.06	0.0591	-0.1606	0.076
Passerines	Both	No	0.5	Intercept	-0.008	-0.0091	0.0667	0.0647	-0.1398	0.1262
				**Habitat (urban)**	**-0.138**	**-0.1383**	**0.0476**	**0.0477**	**-0.2312**	**-0.0431**
				Human footprint	-0.0265	-0.0259	0.0403	0.0394	-0.1061	0.0522
				**Body mass**	**0.0685**	**0.0682**	**0.0196**	**0.0196**	**0.0304**	**0.1077**
				Clutch size	0.0232	0.0233	0.018	0.0179	-0.012	0.0585
				Wing shape	0.0245	0.0245	0.019	0.019	-0.0122	0.0615
				**Migration**	**0.072**	**0.0722**	**0.0238**	**0.0236**	**0.0253**	**0.119**
				Ground foraging	0.0076	0.0075	0.0169	0.017	-0.0248	0.0411
				Flock size	0.0139	0.0139	0.0097	0.0097	-0.0054	0.0329
				**Starting distance**	**0.6987**	**0.6988**	**0.014**	**0.0141**	**0.671**	**0.7261**
				**Season (wet)**	**0.0405**	**0.0406**	**0.0148**	**0.0146**	**0.0123**	**0.0702**
				Tree cover	0.003	0.0028	0.0369	0.036	-0.0683	0.0756
				Continent (Australia)	-0.0088	-0.0093	0.1025	0.1028	-0.2074	0.1914
				Continent (S America)	-0.0507	-0.0518	0.0948	0.0934	-0.2388	0.1373
				Altitude	0.017	0.017	0.0313	0.0316	-0.0441	0.0788
				Latitude	-0.0041	-0.0033	0.0829	0.0814	-0.1688	0.1569
All species	Both	No	0.7	Intercept	0.019	0.0189	0.0489	0.0464	-0.0772	0.1151
				**Habitat (urban)**	**-0.072**	**-0.0719**	**0.0272**	**0.0277**	**-0.1248**	**-0.0186**
				**Human footprint**	**-0.0562**	**-0.0561**	**0.0234**	**0.0231**	**-0.1028**	**-0.0103**
				**Body mass**	**0.1504**	**0.1504**	**0.0139**	**0.0137**	**0.1229**	**0.1774**
				**Clutch size**	**0.0201**	**0.0201**	**0.0096**	**0.0095**	**0.0016**	**0.039**
				**Wing shape**	**0.0406**	**0.0407**	**0.0128**	**0.0126**	**0.0152**	**0.0656**
				Migration	0.0181	0.0181	0.0113	0.0113	-0.0041	0.04
				Ground foraging	0.0067	0.0067	0.0104	0.0104	-0.0138	0.0271
				Flock size	-0.0028	-0.0028	0.0063	0.0062	-0.0152	0.0095
				**Starting distance**	**0.6838**	**0.6838**	**0.0099**	**0.0099**	**0.6645**	**0.703**
				**Season (wet)**	**0.0421**	**0.042**	**0.0086**	**0.0087**	**0.0253**	**0.0593**
				Tree cover	-0.0315	-0.0313	0.0284	0.0281	-0.0874	0.0236
				Continent (Australia)	-0.0975	-0.0967	0.0829	0.0781	-0.2643	0.0642
				Continent (S America)	-0.0057	-0.0064	0.0765	0.0765	-0.1529	0.148
				Altitude	0.0175	0.0174	0.0226	0.0232	-0.026	0.0617
				Latitude	-0.0433	-0.0435	0.0606	0.0596	-0.1598	0.0775
Passerines	Both	No	0.7	Intercept	-0.0078	-0.0086	0.0676	0.0651	-0.14	0.1263
				**Habitat (urban)**	**-0.1408**	**-0.1406**	**0.048**	**0.0475**	**-0.2375**	**-0.0479**
				Human footprint	-0.0263	-0.027	0.0411	0.0407	-0.1064	0.0545
				**Body mass**	**0.0689**	**0.0689**	**0.0201**	**0.0199**	**0.0296**	**0.1079**
				Clutch size	0.0233	0.0231	0.018	0.0185	-0.0113	0.0591
				Wing shape	0.0238	0.024	0.0189	0.0192	-0.0132	0.0607
				**Migration**	**0.0725**	**0.0726**	**0.024**	**0.0243**	**0.0249**	**0.1195**
				Ground foraging	0.0077	0.0082	0.0173	0.0172	-0.0266	0.0414
				Flock size	0.0137	0.0138	0.0097	0.0096	-0.0051	0.0325
				**Starting distance**	**0.7003**	**0.7003**	**0.0139**	**0.0139**	**0.6732**	**0.7275**
				**Season (wet)**	**0.0408**	**0.0406**	**0.0145**	**0.0143**	**0.0128**	**0.0695**
				Tree cover	0.0025	0.0027	0.0367	0.0365	-0.0707	0.0741
				Continent (Australia)	-0.0106	-0.0101	0.1088	0.1077	-0.2264	0.2034
				Continent (S America)	-0.0538	-0.055	0.0986	0.0967	-0.2469	0.1441
				Altitude	0.0179	0.0179	0.0311	0.0312	-0.0426	0.079
				Latitude	-0.0058	-0.0072	0.0879	0.0859	-0.1815	0.1664
All species	Both	No	0.9	Intercept	0.0197	0.0195	0.0503	0.0496	-0.0805	0.1187
				**Habitat (urban)**	**-0.0719**	**-0.0718**	**0.0272**	**0.027**	**-0.1261**	**-0.0188**
				**Human footprint**	**-0.0566**	**-0.0567**	**0.0234**	**0.0233**	**-0.103**	**-0.0116**
				**Body mass**	**0.1503**	**0.1503**	**0.0136**	**0.0136**	**0.1236**	**0.1775**
				**Clutch size**	**0.0201**	**0.0201**	**0.0096**	**0.0095**	**0.0015**	**0.039**
				**Wing shape**	**0.0403**	**0.0403**	**0.0127**	**0.0129**	**0.0156**	**0.0649**
				Migration	0.0181	0.018	0.0114	0.0113	-0.004	0.0404
				Ground foraging	0.0067	0.0069	0.0102	0.0101	-0.0132	0.0271
				Flock size	-0.0028	-0.0027	0.0063	0.0063	-0.0149	0.0097
				**Starting distance**	**0.6843**	**0.6842**	**0.0097**	**0.0097**	**0.6657**	**0.7033**
				**Season (wet)**	**0.0422**	**0.0421**	**0.0086**	**0.0086**	**0.0257**	**0.0591**
				Tree cover	-0.0318	-0.0316	0.0287	0.0288	-0.089	0.0231
				Continent (Australia)	-0.0983	-0.0972	0.085	0.0816	-0.2692	0.0685
				Continent (S America)	-0.0073	-0.0076	0.0782	0.0763	-0.1611	0.1488
				Altitude	0.0176	0.0175	0.023	0.0228	-0.027	0.0627
				Latitude	-0.0456	-0.0453	0.0623	0.0608	-0.17	0.0768

Table S1 continued

**Dataset**			**R^2**	**Variable**	**Mean**	**Median**	**SD**	**MAD**	**2.5% CI**	**97.5% CI**
Passerines	Both	No	0.9	Intercept	-0.0078	-0.0088	0.0671	0.0661	-0.1385	0.1234
				**Habitat (urban)**	**-0.1409**	**-0.1416**	**0.0486**	**0.0488**	**-0.2349**	**-0.0467**
				Human footprint	-0.0261	-0.0263	0.0414	0.0412	-0.1075	0.0534
				**Body mass**	**0.0688**	**0.0691**	**0.0196**	**0.0196**	**0.0299**	**0.1065**
				Clutch size	0.0234	0.0236	0.0178	0.0179	-0.0112	0.0577
				Wing shape	0.024	0.0239	0.0189	0.0185	-0.0132	0.0609
				**Migration**	**0.0726**	**0.0727**	**0.024**	**0.0238**	**0.025**	**0.1188**
				Ground foraging	0.008	0.0079	0.017	0.0171	-0.0253	0.041
				Flock size	0.0137	0.0137	0.0097	0.0098	-0.0052	0.0324
				**Starting distance**	**0.7009**	**0.7008**	**0.0139**	**0.0137**	**0.6737**	**0.7282**
				**Season (wet)**	**0.0409**	**0.0408**	**0.0148**	**0.0145**	**0.0115**	**0.0708**
				Tree cover	0.0027	0.003	0.0374	0.0368	-0.0716	0.0754
				Continent (Australia)	-0.0105	-0.0081	0.1127	0.1127	-0.2343	0.2059
				Continent (S America)	-0.0571	-0.0586	0.1024	0.1017	-0.2591	0.1422
				Altitude	0.0171	0.0167	0.031	0.0305	-0.0428	0.0782
				Latitude	-0.0044	-0.0041	0.0887	0.0867	-0.1758	0.1712

Supplementary Table 1. Results of multivariate Bayesian phylogenetically informed regressions estimating the association between the avian tolerance towards humans (measured as the flight initiation distance; dependent variable) and a set of life-history and environmental variables, for *all species* (N = 10,249 observations for 842 species) and a subset of *passerines* (Order: Passeriformes) (N = 5,400 observations for 425 species). We also fitted our main models using either "habitat type" or human "footprint" (Habitat/footprint) and also subsetting sampled observations only to species that occurred in "both habitats" (Subset). We fitted models with scaled priors for different R^2 values (i.e., R^2 = 0.1, 0.2, 0.3, 0.5, 0.7, and 0.9, respectively). We report the mean and median standardised effect sizes, their standard deviations (SD), median absolute deviations (MAD), and 95% credible intervals (CI). We considered an association significant if the credible intervals did not overlap zero; significant results are highlighted in bold. For details on statistical analyses, see method section in the main text.

References

1. Díaz, M. *et al.* The geography of fear: a latitudinal gradient in anti-predator escape distances of birds across Europe. *PLoS One* **8**, e64634 (2013).
2. Mikula, P. Pedestrian density influences flight distances of urban birds. *Ardea* **102**, 53–60 (2014).
3. Samia, D. S. M., Nakagawa, S., Nomura, F., Rangel, T. F. & Blumstein, D. T. Increased tolerance to humans among disturbed wildlife. *Nat Commun* **6**, 8877 (2015).
4. Thiel, D., Ménoni, E., Brenot, J. F. & Jenni, L. Effects of recreation and hunting on flushing distance of capercaillie. *J Wildl Manage* **71**, 1784–1792 (2007).
5. Weston, M. A., McLeod, E. M., Blumstein, D. T. & Guay, P.-J. A review of flight-initiation distances and their application to managing disturbance to Australian birds. *Emu* **112**, 269–286 (2012).
6. Fox, A. D. & Madsen, J. Behavioural and distributional effects of hunting disturbance on waterbirds in Europe: implications for refuge design. *J Appl Ecol* **34**, 1–13 (1997).
7. Venter, O. *et al.* Last of the Wild Project, Version 3 (LWP-3): 2009 Human Footprint, 2018 Release. *Palisades, New York: NASA Socioeconomic Data and Applications Center (SEDAC)* vol. 7 https://www.nature.com/articles/ncomms12558 (2018).
8. Carrete, M. & Tella, J. L. High individual consistency in fear of humans throughout the adult lifespan of rural and urban burrowing owls. *Sci Rep* **3**, 1–7 (2013).
9. Rebolo-Ifrán, N. *et al.* Links between fear of humans, stress and survival support a non-random distribution of birds among urban and rural habitats. *Sci Rep* **5**, 13723 (2015).
10. Sol, D. *et al.* Risk-taking behavior, urbanization and the pace of life in birds. *Behav Ecol Sociobiol* **72**, 59 (2018).
11. Blumstein, D. T. Developing an evolutionary ecology of fear: how life history and natural history traits affect disturbance tolerance in birds. *Anim Behav* **71**, 389–399 (2006).
12. Mikula, P. *et al.* Adjusting risk-taking to the annual cycle of long-distance migratory birds. *Sci Rep* **8**, 13989 (2018).
13. Samia, D. S. M. & Blumstein, D. T. Birds Flush Early and Avoid the Rush: An Interspecific Study. *PLoS One* **10**, e0119906 (2015).
14. Valcu, M., Dale, J., Griesser, M., Nakagawa, S. & Kempenaers, B. Global gradients of avian longevity support the classic evolutionary theory of ageing. *Ecography* **37**, 930–938 (2014).
15. Wasser, D. E. & Sherman, P. W. Avian longevities and their interpretation under evolutionary theories of senescence. *J Zool* **280**, 103–155 (2010).
16. Stankowich, T. & Blumstein, D. T. Fear in animals: a meta-analysis and review of risk assessment. *Proc R Soc Lond B Biol Sci* **272**, 2627–2634 (2005).
17. Wilman, H. *et al.* EltonTraits 1.0: Species-level foraging attributes of the world's birds and mammals. *Ecology* **95**, 2027–2027 (2014).
18. Dunning, J. B. *CRC Handbook of Avian Body Masses*. (CRC Press, 2008).
19. del Hoyo, J., Elliott, A., Sargatal, J., Christie, D. A. & Kirwan, G. *Handbook of the Birds of the World Alive*. (Lynx Edicions, 2020).
20. Møller, A. P. & Garamszegi, L. Z. Between individual variation in risk-taking behavior and its life history consequences. *Behav Ecol* **23**, 843–853 (2012).
21. Samia, D. S. M., Møller, A. P. & Blumstein, D. T. Brain size as a driver of avian escape strategy. *Sci Rep* **5**, 11913 (2015).
22. Ghalambor, C. K. & Martin, T. E. Fecundity-survival trade-offs and parental risk-taking in birds. *Science* **292**, 494–497 (2001).
23. Payne, R. B. Individual laying histories and the clutch size and numbers of eggs of parasitic cuckoos. *Condor* **75**, 414 (1973).
24. Jetz, W., Sekercioglu, C. H., Böhning-Gaese, K., Burgess, N. & Powell, G. The worldwide variation in avian clutch size across species and space. *PLoS Biol* **6**, e303 (2008).

25. Cariello, M. O., Lima, M. R., Schwabl, H. G. & Macedo, R. H. Egg characteristics are unreliable in determining maternity in communal clutches of guira cuckoos Guira guira. *J Avian Biol* **35**, 117–124 (2004).
26. Mark, M. M. & Rubenstein, D. R. Physiological costs and carry-over effects of avian interspecific brood parasitism influence reproductive tradeoffs. *Horm Behav* **63**, 717–722 (2013).
27. Reynolds, C., Henry, D. A. W., Tye, D. R. C. & Tye, N. D. Defining separation zones for coastal birds at a wetland of global importance. *Wildlife Research* **48**, 134–141 (2020).
28. Mikula, P. *et al.* Migratory and resident waders differ in risk taking on the wintering grounds. *Behavioural Processes* **157**, 309–314 (2018).
29. Birdlife International. Data Zone. http://datazone.birdlife.org/home (2020).
30. Møller, A. P., Vágási, C. I. & Pap, P. L. Risk-taking and the evolution of mechanisms for rapid escape from predators. *J Evol Biol* **26**, 1143–1150 (2013).
31. Lockwood, R., Swaddle, J. P. & Rayner, J. M. v. Avian wingtip shape reconsidered: Wingtip shape indices and morphological adaptations to migration. *J Avian Biol* **29**, 292 (1998).
32. Claramunt, S., Derryberry, E. P., Remsen, J. v. & Brumfield, R. T. High dispersal ability inhibits speciation in a continental radiation of passerine birds. *Proc R Soc Lond B Biol Sci* **279**, 1567–1574 (2012).
33. Pigot, A. L. & Tobias, J. A. Dispersal and the transition to sympatry in vertebrates. *Proc R Soc Lond B Biol Sci* **282**, (2015).
34. Sheard, C. *et al.* Ecological drivers of global gradients in avian dispersal inferred from wing morphology. *Nat Commun* **11**, 2463 (2020).
35. Fernández-Juricic, E., Gall, M. D., Dolan, T., Tisdale, V. & Martin, G. R. The visual fields of two ground-foraging birds, House Finches and House Sparrows, allow for simultaneous foraging and anti-predator vigilance. *Ibis* **150**, 779–787 (2008).
36. Moore, B. A., Pita, D., Tyrrell, L. P. & Fernández-Juricic, E. Vision in avian emberizid foragers: Maximizing both binocular vision and fronto-lateral visual acuity. *J Exp Biol* **218**, 1347–1358 (2015).
37. Fernández-Juricic, E. *et al.* Visual systems and vigilance behaviour of two ground-foraging avian prey species: White-crowned sparrows and California towhees. *Anim Behav* **81**, 705–713 (2011).
38. Sorato, E., Gullett, P. R., Griffith, S. C. & Russell, A. F. Effects of predation risk on foraging behaviour and group size: Adaptations in a social cooperative species. *Anim Behav* **84**, 823–834 (2012).
39. Götmark, F. & Post, peter. Prey selection by sparrowhawks, *Accipiter nisus*: Relative predation risk for breeding passerine birds in relation to their size, ecology and behaviour. *Philos Trans R Soc B* **351**, 1559–1577 (1996).
40. Blumstein, D. T., Fernández-Juricic, E., Zollner, P. A. & Garity, S. C. Inter-specific variation in avian responses to human disturbance. *J Appl Ecol* **42**, 943–953 (2005).
41. Carrascal, L. M. & Alonso, C. L. Habitat use under latent predation risk. A case study with wintering forest birds. *Oikos* **112**, 51–62 (2006).
42. Pulliam, H. On the advantages of flocking. *J Theor Biol* **38**, 419–422 (1973).
43. Roberts, G. Why individual vigilance declines as group size increases. *Anim Behav* **51**, 1077–1086 (1996).
44. Morelli, F. *et al.* Contagious fear: Escape behavior increases with flock size in European gregarious birds. *Ecol Evol* **9**, 6096–6104 (2019).
45. Tryjanowski, P., Kosicki, J. Z., Hromada, M. & Mikula, P. The emergence of tolerance of human disturbance in Neotropical birds. *J Trop Ecol* **36**, 1–5 (2020).
46. Laursen, K., Kahlert, J. & Frikke, J. Factors affecting escape distances of staging waterbirds. *Wildlife Biol* **11**, 13–19 (2005).
47. Linley, G. D. *et al.* Are disturbance separation distances derived from single species applicable to mixed-species shorebird flocks? *Wildl Res* **46**, 719–723 (2019).

48. Cox, D. T. C. *et al.* The seasonality of breeding in savannah birds of West Africa assessed from brood patch and juvenile occurrence. *J Ornithol* **154**, 671–683 (2013).
49. Hau, M. Timing of breeding in variable environments: Tropical birds as model systems. *Horm Behav* **40**, 281–290 (2001).
50. Stouffer, P. C., Johnson, E. I. & Bierregaard Jr., R. O. Breeding Seasonality in Central Amazonian Rainforest Birds. *Auk* **130**, 529–540 (2013).
51. Wyndham, E. Length of birds' breeding seasons. *American Naturalist* **128**, 155–164 (1986).
52. Poulin, B., Lefebvre, G. & McNeil, R. Tropical avian phenology in relation to abundance and exploitation of food resources. *Ecology* **73**, 2295–2309 (1992).
53. Araujo, H. F. P. de, Vieira-Filho, A. H., Barbosa, M. R. de v., Diniz-Filho, J. A. F. & Silva, J. M. C. da. Passerine phenology in the largest tropical dry forest of South America: effects of climate and resource availability. *Emu* **117**, 78–91 (2017).
54. Doughty, C. E., Faurby, S. & Svenning, J.-C. The impact of the megafauna extinctions on savanna woody cover in South America. *Ecography* **39**, 213–222 (2016).
55. Lehmann, C. E. R. *et al.* Savanna vegetation-fire-climate relationships differ among continents. *Science* **343**, 548–552 (2014).
56. Braimoh, B. *et al.* Managing human disturbance: factors influencing flight-initiation distance of birds in a West African nature reserve. *Ostrich* **89**, 59–69 (2018).
57. Global Forest Watch. Global Forest Change. https://data.globalforestwatch.org/ (2020).
58. Møller, A. P., Samia, D. S. M., Weston, M. A., Guay, P.-J. & Blumstein, D. T. American exceptionalism: population trends and flight initiation distances in birds from three continents. *PLoS One* **9**, e107883 (2014).
59. Møller, A. P. & Liang, W. Tropical birds take small risks. *Behav Ecol* **24**, 267–272 (2013).
60. Wiersma, P., Muñoz-Garcia, A., Walker, A. & Williams, J. B. Tropical birds have a slow pace of life. *Proc Natl Acad Sci U S A* **104**, 9340–9345 (2007).
61. Ricklefs, R. E. & Wikelski, M. The physiology/life-history nexus. *Trends Ecol Evol* **17**, 462–468 (2002).
62. Andrade, M. & Blumstein, D. T. Anti-predator behavior along elevational and latitudinal gradients in dark-eyed juncos. *Curr Zool* **66**, 239–245 (2020).
63. Ekanayake, K. B. *et al.* Ecological and environmental predictors of escape among birds on a large tropical island. *Behav Ecol Sociobiol* **76**, 1–13 (2022).
