# Supplementary material for: Bird tolerance to humans in open tropical ecosystems
Source: Nat Commun. 2023 Apr 20;14:2146. doi: 10.1038/s41467-023-37936-5 (PMC10119130; doi:10.1038/s41467-023-37936-5)
Supplement: Supplementary file 3 — Reporting Summary [file 41467_2023_37936_MOESM3_ESM.pdf]

nature portfolio

Corresponding author(s): Peter Mikula

Last updated by author(s): Feb 18, 2023

Reporting Summary

Nature Portfolio wishes to improve the reproducibility of the work that we publish. This form provides structure for consistency and transparency in reporting. For further information on Nature Portfolio policies, see our Editorial Policies and the Editorial Policy Checklist.

Statistics

For all statistical analyses, confirm that the following items are present in the figure legend, table legend, main text, or Methods section.

n/a	Confirmed	
☐	☒	The exact sample size (*n*) for each experimental group/condition, given as a discrete number and unit of measurement
☐	☒	A statement on whether measurements were taken from distinct samples or whether the same sample was measured repeatedly
☐	☒	The statistical test(s) used AND whether they are one- or two-sided<br>*Only common tests should be described solely by name; describe more complex techniques in the Methods section.*
☐	☒	A description of all covariates tested
☐	☒	A description of any assumptions or corrections, such as tests of normality and adjustment for multiple comparisons
☐	☒	A full description of the statistical parameters including central tendency (e.g. means) or other basic estimates (e.g. regression coefficient) AND variation (e.g. standard deviation) or associated estimates of uncertainty (e.g. confidence intervals)
☐	☒	For null hypothesis testing, the test statistic (e.g. *F*, *t*, *r*) with confidence intervals, effect sizes, degrees of freedom and *P* value noted<br>*Give P values as exact values whenever suitable.*
☐	☒	For Bayesian analysis, information on the choice of priors and Markov chain Monte Carlo settings
☐	☒	For hierarchical and complex designs, identification of the appropriate level for tests and full reporting of outcomes
☐	☒	Estimates of effect sizes (e.g. Cohen's *d*, Pearson's *r*), indicating how they were calculated

Our web collection on statistics for biologists contains articles on many of the points above.

Software and code

Policy information about availability of computer code

Data collection	Data collection was done by the authors of the study. No special software or code was used.
Data analysis	All statistical analyses were performed in open software R (version 4.1.2) and the following packages: phangorn (version 2.8.1), cmdstanr (version 0.4.0), and posterior (version 1.1.0). The code used to generate the results can be found at the Open Science Framework repository (https://doi.org/10.17605/OSF.IO/BSPQX).

For manuscripts utilizing custom algorithms or software that are central to the research but not yet described in published literature, software must be made available to editors and reviewers. We strongly encourage code deposition in a community repository (e.g. GitHub). See the Nature Portfolio guidelines for submitting code & software for further information.

Data

Policy information about availability of data

All manuscripts must include a data availability statement. This statement should provide the following information, where applicable:

- Accession codes, unique identifiers, or web links for publicly available datasets
- A description of any restrictions on data availability
- For clinical datasets or third party data, please ensure that the statement adheres to our policy

Source data are provided along this papers. Data on flight initiation distance, starting distance, flock size, habitat type, and geographic position were collected by the authors of this study. Data on body mass were extracted from EltonTraits 1.0 database56; on clutch sizes from Handbook of the Birds of the World Alive; hand-wing index from Sheard et al. (2020; Nat Commun 11, 2463); migratory behaviour from BirdLife's database; wet season from the Climate Change Knowledge Portal

(https://climateknowledgeportal.worldbank.org); ground foraging from EltonTraits. Data on tree cover (available at https://data.globalforestwatch.org/)59, altitude (available at https://earthexplorer.usgs.gov/; United States Geological Survey), and 2009 human footprint index (available at https://sedac.ciesin.columbia.edu/data/set/wildareas-v3-2009-human-footprint) were extracted using ArcGIS and associated extensions and toolboxes (e.g. Spatial Analyst). The data supporting the results and the supplementary information of this study can be found at the Open Science Framework repository (https://doi.org/10.17605/OSF.IO/BSPQX).

Human research participants

Policy information about studies involving human research participants and Sex and Gender in Research.

Reporting on sex and gender	We did not study humans.
Population characteristics	We did not study humans.
Recruitment	We did not study humans.
Ethics oversight	We did not study humans.

Note that full information on the approval of the study protocol must also be provided in the manuscript.

Field-specific reporting

Please select the one below that is the best fit for your research. If you are not sure, read the appropriate sections before making your selection.

☐ Life sciences ☐ Behavioural & social sciences ☒ Ecological, evolutionary & environmental sciences

For a reference copy of the document with all sections, see nature.com/documents/nr-reporting-summary-flat.pdf

Ecological, evolutionary & environmental sciences study design

All studies must disclose on these points even when the disclosure is negative.

Study description	We aimed to identify a key life-history, ecological and environmental traits that best predict direction and magnitude of tolerance of savannah birds towards humans (measured as flight initiation distance) by using Bayesian phylogenetically- and spatially-informed analyses.
Research sample	Our sample covers birds (class Aves) inhabiting open tropical ecosystems of three continents (Africa, South America, Australia) for which data on escape distances, life history, ecology and other traits were available. We tried to cover as much sites in open tropical ecosystems as possible. Moreover, at most sampling sites, we sampled as much bird species and individuals as possible. We generally did not focus on population of particular species.<br><br>Altogether, we collected 14,998 flight initiation distances for 953 bird species (120 families and 32 orders). However, this sample was reduced to 10,249 observations for 842 species (in full dataset) and 5,400 observations for 425 species (in dataset for passerines), respectively, because some predictor values were missing for some species. For details on sampled localities, populations, and species, see information at the Open Science Framework repository (https://doi.org/10.17605/OSF.IO/BSPQX).
Sampling strategy	We were primarily interested in escape behaviour of birds when facing approaching human. Because this type of behaviour is context, population, and species-specific, we put together data from as sites, populations, and species as possible. The sample sizes in the final analyses were determined by the data availability for used variables.
Data collection	Data on flight initiation distance, starting distance, flock size, habitat type, and site position were collected directly in the field by the authors of the study. For further details and individual-based measurements, see information at the Open Science Framework repository (https://doi.org/10.17605/OSF.IO/BSPQX); each sampled bird individual is assigned to a name of observer. Escape distances were estimated by a number of ~1 m steps, conversion of a number of steps to metres, or using a rangefinder. Field data were then typically written down to the field notepad.<br>Data on body mass, clutch size, wing shape, migratory behaviour, and ground foraging were extracted from the literature by PM. Data on season, tree cover and altitude were extracted using open online tools and ArcGIS by DR.
Timing and spatial scale	In Africa, the fieldwork was conducted between 2002 and 2021; African sampling spanned much of the continent, from Senegal and Mauritania in the West, to Kenya and South Africa in the East and South, respectively. In South America, data were collected between 2011 and 2021 in the Cerrado and Pantanal ecoregions (Brazil), and the Llanos ecoregion (Colombia). In Australia, data were collected in 2000 and again between 2011 and 2017 in Queensland and in the Northern Territory.<br><br>Altogether, data sampling was circumtropical, covering Australia, South America (Brazil, Colombia), and several countries in Africa (Benin, Burkina Faso, Ethiopia, Ghana, Chad, Ivory Coast, Kenya, Mali, Mauritania, Namibia, Niger, Nigeria, Senegal, South Africa, Sudan, Tanzania, Uganda, Zambia and Zimbabwe).
Data exclusions	No data has been excluded from the study.
Reproducibility	Previous studies found that flight initiation distance is highly consistent for individuals, populations and species under similar

contexts. Because this type of behaviour is context, population, and species-specific, we put together data from as sites, populations, and species as possible. To improve robustness of our results, we sampled as much bird species and individuals as possible at most sites. All field data were collected using previously published protocols, briefly described also in a method section of this study. All raw data are deposited at the Open Science Framework repository (https://doi.org/10.17605/OSF.IO/BSPQX).

Randomization	We took into account phylogenetic, spatial, temporal, and collector non-independence by always including phylogeny, spatial covariance matrix and species, site ID, year and collector ID as a random factors in regression models.
Blinding	Most data were collected before this study started, i.e. were collected blindly regarding hypotheses tested in the present study.

Did the study involve field work? [x] Yes [ ] No

Field work, collection and transport

Field conditions	All field data were collected in open tropical ecosystems of three continents (Africa, South America, Australia). The most of data were gathered in the morning (06:00–10:00) and evening (15:00–18:00) when birds are most active. Field data were collected during favourable weather, mainly during sunny days with no or weak wind. We excluded observations from large patches of habitats markedly differing in vegetation structure from open tropical habitats, such as closed forests.
Location	Our fieldwork was conducted in one country in South America (Brazil, Colombia), Australia and several countries in Africa (Benin, Burkina Faso, Ethiopia, Ghana, Chad, Ivory Coast, Kenya, Mali, Mauritania, Namibia, Niger, Nigeria, Senegal, South Africa, Sudan, Tanzania, Uganda, Zambia and Zimbabwe). For details on sampled localities, populations, and species, see information at the Open Science Framework repository (https://doi.org/10.17605/OSF.IO/BSPQX).
Access & import/export	No import/export of samples was required to complete this study.
Disturbance	All fieldwork was conducted in accordance with the approved guidelines. Data were collected in public places and on private lands where no special permit was required. The method used to estimate avian tolerance towards human disturbance was designed to cause only brief and minimal disturbance to birds; in cities, this disturbance typically does not differ from standard background disturbance caused by other site visitors.

Reporting for specific materials, systems and methods

We require information from authors about some types of materials, experimental systems and methods used in many studies. Here, indicate whether each material, system or method listed is relevant to your study. If you are not sure if a list item applies to your research, read the appropriate section before selecting a response.

Materials & experimental systems

n/a	Involved in the study	
[x]	[ ]	Antibodies
[x]	[ ]	Eukaryotic cell lines
[x]	[ ]	Palaeontology and archaeology
[ ]	[x]	Animals and other organisms
[x]	[ ]	Clinical data
[x]	[ ]	Dual use research of concern

Methods

n/a	Involved in the study	
[x]	[ ]	ChIP-seq
[x]	[ ]	Flow cytometry
[x]	[ ]	MRI-based neuroimaging

Animals and other research organisms

Policy information about studies involving animals; ARRIVE guidelines recommended for reporting animal research, and Sex and Gender in Research

Laboratory animals	No laboratory animals have been used in this study.
Wild animals	Our study was observational and included 953 species of birds.
Reporting on sex	We did not collect data on the sex of animals.
Field-collected samples	Our study was observational and did not include samples collected from the field.
Ethics oversight	In Kenya, field data collection was approved by National Commission for Science Technology and Innovation no. NACOSTI/P14/4653/660 and no. NACOSTI/P18/52438/25493, Kenya Wildlife Service no KWS/BRP/5001. In South Africa, the University of Cape Town Science Faculty Animal Ethics Committee (2015/V11/SC), Northern Cape Department of Environment and Nature Conservation (FAUNA 1489/2015). In Brazil, we worked on private lands where no permits were not required. In Australia, research was approved

by the Deakin University (B32/2012, B11/2015, B10/2018), the Charles Darwin University Animal Ethics (A11013), the Macquarie University Animal Research Committee (99021), the Queensland Parks and Wildlife Service (#FA/000379/00/SA), and the Northern Territory Parks and Wildlife (41035 and 55233).

Note that full information on the approval of the study protocol must also be provided in the manuscript.
